# scMoC: single-cell multi-omics clustering

**DOI:** 10.1093/bioadv/vbac011

**Published:** 2022-02-15

**Authors:** Mostafa Eltager, Tamim Abdelaal, Ahmed Mahfouz, Marcel J T Reinders

**Affiliations:** 1 Delft Bioinformatics Lab, Delft University of Technology, Delft 2628XE, The Netherlands; 2 Leiden Computational Biology Center, Leiden University Medical Center, Leiden 2333ZC, The Netherlands; 3 Division of Image Processing, Department of Radiology, Leiden University Medical Center, Leiden, 2333ZC, The Netherlands; 4 Department of Human Genetics, Leiden University Medical Center, Leiden 2333ZC, The Netherlands

## Abstract

**Motivation:**

Single-cell multi-omics assays simultaneously measure different molecular features from the same cell. A key question is how to benefit from the complementary data available and perform cross-modal clustering of cells.

**Results:**

We propose **S**ingle-**C**ell **M**ulti-**o**mics **C**lustering (scMoC), an approach to identify cell clusters from data with comeasurements of scRNA-seq and scATAC-seq from the same cell. We overcome the high sparsity of the scATAC-seq data by using an imputation strategy that exploits the less-sparse scRNA-seq data available from the same cell. Subsequently, scMoC identifies clusters of cells by merging clusterings derived from both data domains individually. We tested scMoC on datasets generated using different protocols with variable data sparsity levels. We show that scMoC (i) is able to generate informative scATAC-seq data due to its RNA-guided imputation strategy and (ii) results in integrated clusters based on both RNA and ATAC information that are biologically meaningful either from the RNA or from the ATAC perspective.

**Availability and implementation:**

The data used in this manuscript is publicly available, and we refer to the original manuscript for their description and availability. For convience sci-CAR data is available at NCBI GEO under the accession number of GSE117089. SNARE-seq data is available at NCBI GEO under the accession number of GSE126074. The 10X multiome data is available at the following link https://www.10xgenomics.com/resources/datasets/pbmc-from-a-healthy-donor-no-cell-sorting-3-k-1-standard-2-0-0.

**Supplementary information:**

Supplementary data are available at *Bioinformatics Advances* online.

## 1 Introduction

Recent developments in single-cell technologies have enabled measuring different molecular features with increasing throughput. However, due to the destructive nature of most protocols, these molecular features can be measured from the same biological sample but from different cells. Recent advances have made it possible to profile more than one ‘omic’ data from the same cell, introducing single-cell multimodal omics (Zhu *et al.*, 2020). Such technology enables simultaneous measurement of gene expression through **s**ingle-cell transcriptome sequencing (scRNA-seq) and chromatin accessibility through single-cell transposase-accessible chromatin sequencing (scATAC-seq; Cao *et al.*, 2018; Chen *et al.*, 2019b). It is anticipated that single-cell multimodal omics is a promising technology that will improve our ability to dissect the complex gene regulatory networks, cell lineages and trajectories (Schier, 2020).

Several methods are proposed to integrate different omics data from unpaired datasets, i.e. measured from different cells but from the same biological sample. For example, Seurat V3 (Stuart *et al.*, 2019) and LIGER (Welch *et al.*, 2019) map the common feature space into an aligned latent domain using dimensionality reduction. Nevertheless, single-cell multimodal omics brings up a different computational challenge by offering *paired data* points for the same cell (Ma *et al.*, 2020; Stuart and Satija, 2019; Zhu *et al.*, 2020).

One of the main challenges lies in the data sparsity, which is the percentage of the observed zeros in the data. For example, the data density (percentage of nonzeros in the data) in unimodal scRNA-seq datasets usually ranges from 10% to 45% and from 1% to 10% in unimodal scATAC-seq datasets (Chen *et al.*, 2019a). However, in multimodal omics measurements, these densities are considerably lower (1–10% for the scRNA-seq data and 0.2–6% for scATAC-seq) due to the prematurity of the protocols (Stuart and Satija, 2019). Although there is a higher chance to observe a zero measurement from genes with low expression levels (Ntranos *et al.*, 2019), higher data sparsity can deteriorate cluster separation and, thus, make it hard to capture a clear structure in the cellular composition (Baek and Lee, 2020; Luecken *et al.*, 2020).

The cluster agreement between data from different modalities represents another challenge. Although those measurements of different modalities are taken from the same cells, they reflect different functionalities within a cell. Hence, when approached individually, these measurements will not result in the same grouping of cells, complicating the decision on a correct grouping as well as the establishment of cell types. So, this calls for algorithms that can exploit the complementary view on the same cell (Stuart and Satija, 2019).

To tackle these challenges, we propose **S**ingle-**C**ell **M**ulti-**o**mics **C**lustering (scMoC). scMoC is designed to cluster paired multimodal datasets that measure both single-cell transcriptomics sequencing (scRNA-seq) and single-cell transposase accessibility chromatin sequencing (scATAC-seq). The most important ingredient of scMoC is that it imputes the sparse scATAC-seq data using an RNA-guided imputation process. A *k-nn*-based imputation imputes ATAC peak counts for a specific cell from a set of similar cells, by the average of the peak counts found in the set of similar cells. This imputation scheme relies on the set of similar cells, which are hard to find when data sparsity is high as that impacts the resemblance of peak profiles. By recognizing that we have measured RNA counts from the same cells and that the RNA data sparsity is much lower, we propose to calculate cell similarities not in the ATAC domain, but in the RNA domain. We show that the resulting RNA-guided imputed ATAC data are better structured and provide informative and complementary data in comparison to the analysis based on the RNA data only.

## 2 Methods

### 2.1 Single-Cell Multi-omics Clustering

**Fig. 1.**
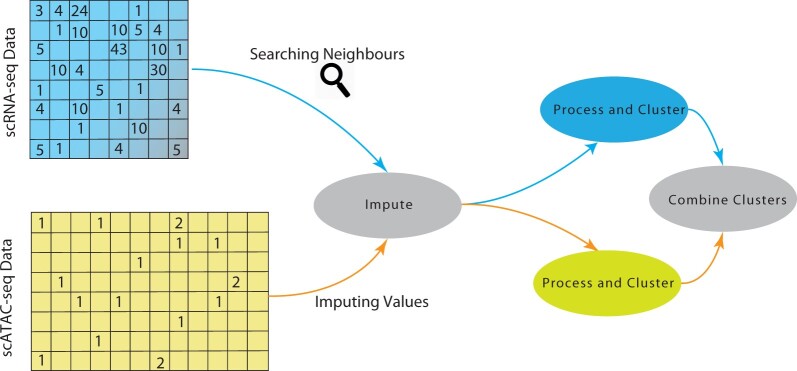
Schematic overview of scMoC. scMoC clusters multimodal single-cell data based on scRNA-seq and scATAC-seq measurements from the same cell. It encompasses an RNA-guided imputation strategy to leverage the higher data sparsity of the scRNA-seq data (with respect to the scATAC-seq data). scMoC builds on the idea that cell–cell similarities can be better estimated from the RNA profiles and then used to define a neighborhood to impute from it the ATAC data, since these are comeasured from the same cell. After the imputation, the two modalities are clustered individually and then combined into one clustering in which RNA-based clusters are being split if there is enough evidence from the ATAC data

A general overview of scMoC is shown in Figure 1. Briefly, to overcome the data sparsity in the scATAC-seq data, we impute the scATAC-seq data. Here, we use the fact that the data are paired and guide the scATAC-seq imputation by choosing similar cells based on the RNA measurements of the cell. Next, we cluster the scRNA-seq and the imputed scATAC-seq independently and merge the resulting clusters. In the following sections, we describe the details of the method. A more precise workflow can be found in Supplementary Figure 1.

#### 2.1.1 Preprocessing of the data

scRNA-seq data are preprocessed following the Seurat 3.0 pipeline (Stuart *et al.*, 2019). For the scRNA-seq data, first, a quality control (QC) step is performed to remove noisy and almost empty cells. These thresholds are database dependent (Section 2.2 and Supplementary Table S1). scRNA-seq counts are normalized by dividing each count by the total count for each cell and then scaling up using a factor of 1e4, followed by a $\log\left(x+1\right)$ transformation. The top 6000 highly variable genes are selected, using the variance stabilizing transform (vst) algorithm. Subsequently, the data are centered around zero and scaled by dividing each expression by the standard deviation. Next, we projected the data on the top 20 principal components for further processing. The choice of the parameters is based on our empirical experience with the data or on the commonly used practices and default parameters.

For the scATAC-seq data, we followed common pipeline steps (Baek and Lee, 2020). First, we removed almost empty and noisy cells (Dataset and Supplementary Table S1). Peak counts are normalized by dividing each count by the total count for each cell and then scaling up using a factor of 1e4. This is followed by an imputation step (next section). After imputation, the scATAC-seq data are renormalized and $\log\left(x+1\right)$ transformed and then scaled. The Latent Semantic Indexing (LSI) is used to reduce the dimensionality of the scATAC-seq data as applied in previous studies (Cusanovich *et al.*, 2018; Chen *et al.*, 2018). Also, we have experimented the effect of using LSI versus using PCA (Supplementary Fig. S2). However, the difference is not major, it is in favor of LSI usage. So, the top 20 LSI vectors are used for further processing.

#### 2.1.2 Imputation of the scATAC-seq data

To deal with the sparsity of the scATAC-seq data, we resort to an imputation strategy. Here, we exploit the fact that for the same cell we have RNA and ATAC measurements. Hence, we propose to guide the imputation of the scATAC-seq data using the scRNA-seq data because cell-cell similarities can be calculated more robustly on the scRNA-seq data as it suffers less from data sparsity. For this *RNA-guided imputation*, a set of similar cells is chosen for each cell. Hereto, we choose the top-*k* (*k* = 50 throughout this work) closest cells based on the Euclidean distance between cells in the PCA projected space of the RNA measurements. scATAC-seq peak values are then imputed by taking the average peak value across the *k*-nearest neighboring cells.

For reference, we also considered a *self-imputation* in which the *k*-nearest neighboring cells are selected on the basis of the scATAC-seq data only, using the Euclidean distance of the unimputed scATAC-seq data projected in the LSI space as a measurement of the distance between cells. The imputation method afterwards remains the same as mentioned in the RNA-guided imputation.

#### 2.1.3 Unimodal clustering

Both, the scRNA-seq and (imputed) scATAC-seq data are clustered separately using the Louvain algorithm (Blondel *et al.*, 2008), implemented in Seurat with the default graph resolution of 0.8. To build the neighborhood graph, we used the *k*-nearest neighbor algorithm (with *k* = 50) based on the Euclidean distance.

#### 2.1.4 Combining RNA and ATAC clustering

To combine the scRNA-seq and (imputed) scATAC-seq based clusters, we (again) use a strategy that trusts the scRNA-seq rich data more than the scATAC-seq data. Hence, we choose only to split RNA-based clusters when there is enough evidence from the scATAC-seq data (and not the other way around). To do so, we construct a contingency table representing the percentage of agreement between both clustering. When an RNA cluster overlaps with more than one ATAC cluster, scMoC splits the RNA cluster accordingly, i.e. if the overlapping percentage of the ATAC cluster is >10% and <90% of the RNA cluster, we accept that split. After splitting, the nonassigned cells from the original RNA cluster are assigned to the closest cluster based on the average distance to the cells within a cluster using Euclidean distance in the RNA space only.

For reference, we also experimented to do the imputation with an *RNAcluster guided imputation*; i.e. limiting the neighborhood search to the cells in the matching RNA cluster. This method shows a deteriorated performance, especially the Uniform Manifold Approximation and Projection for dimension reduction (UMAP) plots show a scattered and line-like structure. Results are shown in Supplementary Figure S3.

### 2.2 Datasets

Three different datasets were used to test scMoC (summarized in Table 1): *mouse kidney data*, measured using the sci-CAR protocol (Cao *et al.* 2018); *adult mouse cerebral cortex data*, measured using the SNARE-seq protocol (Chen *et al.* 2019b); and *human peripheral blood mononuclear cells (PBMC) data* from 10X genomics Multiome. These datasets were chosen because they range in their data density; about 6-fold for the scRNA-seq data and more than 20-fold for the ATAC data. For the QC step, each dataset is filtered differently based on the QC metric visualization tools provided in Seurat package (Stuart *et al.* 2019). Supplementary Table S1 lists the limits used in this step. Supplementary Table S2 indicates the numbers and percentages of cells passed the QC step for each dataset, confirming that the QC steps did not result in a significant reduction in the number of cells in both modalities.

**Table 1.** Description of the datasets used in this study

	sci-CAR	SNARE-seq	10X genomics
Tissue	Mouse kidney	Mouse brain	PBMC
No. of cells	11 296	10 309	3003
No. of genes (RNA)	49 584	33 160	21 547
No. of peaks (ATAC)	252 741	244 544	75 540
RNA data density	1.05%	2.87%	7.06%
ATAC data density	0.28%	1.01%	6.49%

### 2.3 Downstream analysis

The list of well-known marker genes for cell types is taken from http://bio-bigdata.hrbmu.edu.cn/CellMarker/. Mean expression of each marker is inspected for each cluster.

Differentially expressed (DE) genes for each cluster with respect to all other clusters are done using the Wilcoxon rank-sum test with Bonferroni for multiple test correction. To do so, we used *FindAllMarkers* function from Seurat V3 with default parameters except for the minimum percentage (min.pct) which is set to 0.25 and tested only positive markers (set *only.pos* to True). The list of the DE genes is then intersected with the list of the well-known marker genes to get the common genes between the two lists to identify the cell clusters.

For each cluster, an enrichment test for the known motifs is done using *FindMotif* function from Signac package (Stuart *et al.*, 2020) using the default setup and parameters.

## 3 Results

### 3.1 scMoC reveals new cell clusters from the data

#### 3.1.1 RNA-guided imputation improves ATAC data quality

**Fig. 2.**
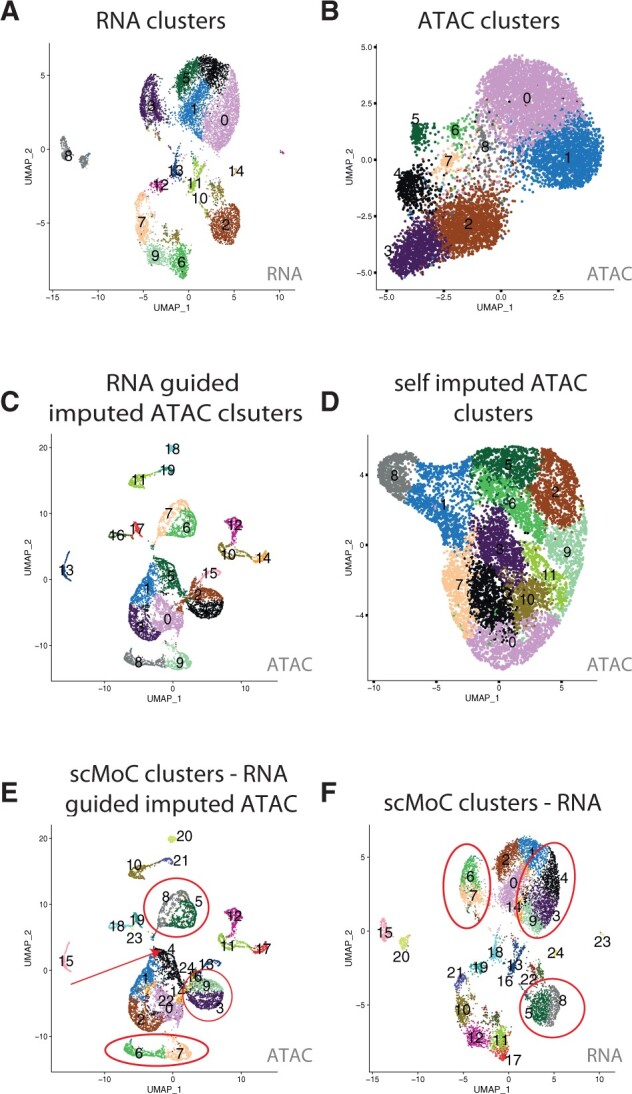
scMoC shows different cluster in the scRNA-seq data based on the imputed scATAC-seq data. UMAP visualizations of the sci-CAR. (**A**) scRNA-seq data, colors indicating RNA-based clusters. (**B**) scATAC-seq data, colors indicating ATAC-based clusters. (**C**) RNA-guided imputed scATAC-seq data, colors indicating clusters within the imputed data. (**D**) Self-imputed scATAC-seq data, colors indicating clusters detected in these data. (**E**) RNA-guided imputed scATAC-seq data using the scMoC clusters. (**F**) scRNA-seq data using the scMoC clusters. Clusters in both E and F are named and colored identically according to scMoC clusters

We tested scMoC on the sci-CAR dataset. Figure 2A shows the UMAP for the scRNA-seq part of the data only as well as the identified RNA-based clusters (Section 2), which shows that the clusters of cells are clearly separable. This is not the case for the scATAC-seq part of the data (Fig. 2B), also resulting in low cluster agreement between the two clusterings (Supplementary Fig. S4). Probably that is driven by the high sparsity in the scATAC-seq data (99.7% zeros, see Table 1), largely influencing distances between cells in the ATAC data. This can be resolved by imputing the ATAC data. Hereto, we applied our proposed RNA-guided imputation (Section 2), which decreased the data sparsity to 89%. The resulting imputed ATAC data indeed shows well-separated clusters (Fig. 2C), in addition to better agreement with RNA-based clusters (Supplementary Fig. S5). For comparison, we imputed the ATAC data with the self-imputed scheme (using only the ATAC data, Section 2), which slightly reduced the data sparsity (95.9%) but did not result in separable clusters (Fig. 2D), as well as a low cluster agreement with the RNA-based clusters (Supplementary Fig. S6).

#### 3.1.2 ATAC-based clusters refine RNA-based cluster

Next, we combined the RNA-guided imputed ATAC-based clusters with the RNA-based clusters using scMoC (Section 2). Figure 2E and F shows the resulting scMoC clusters overlaid on the UMAPs of the RNA-guided imputed scATAC-seq data and scRNA-seq, respectively. This shows that the original RNA-based clusters are split based on the scATAC-seq data. For example, the red circles in Figure 2F indicate splits of the RNA-based clustering due to the available ATAC data of these same cells.

#### 3.1.3 ATAC refined clusters provide complementary information

To ensure that scMoC clusters cannot be achieved just by refining the clustering resolution, we compared the scMoC clusters to more fine-grained clusterings of, both, the scRNA-seq and the imputed scATAC-seq data. Supplementary Figure S7 shows how different clusterings are related. For example, scMoC cluster 22 (scMoC_22) overlaps with scRNA-seq cluster 27 (RNA_27) as well as the scATAC-seq based cluster 33 (ATAC_33), indicating an agreement of the grouping of cells when looking only at RNA or ATAC-based information. Alternatively, scMoC cluster 8 (scMoC_8) overlaps with scRNA-seq cluster 6 (RNA_6) and combines two scATAC-seq-based clusters (ATAC_16, ATAC_28). This nicely shows that the ATAC data provide additional information to split the RNA cluster. Interestingly more complex combinations do occur. For example, scMoC cluster 0 (scMoC_0) combines two scRNA-seq clusters (RNA_2 and RNA_7) and (parts of) four scATAC-seq-based clusters (ATAC_2, ATAC_3 ATAC_15 and ATAC_18). This complex combination is further supported by the contingency table between the scRNA-seq and RNA-guided imputed scATAC-seq clusters as there is no one-to-one agreement between these clusters (Supplementary Fig. S8). To ensure that these clusters are not generated due to differences in the quality of assays, we overlayed the QC matrices on the UMAP for both scRNA-seq and scATAC-seq data in Supplementary Figure S9. The UMAPs show a homogeneous distribution for the cell qualities suggesting that clusters are not related to the variability in cell qualities.

### 3.2 Biological interpretation for scMoC clusters

#### 3.2.1 Genes differentiating scMoC clusters overlap with cell-type marker genes

**Fig. 3.**
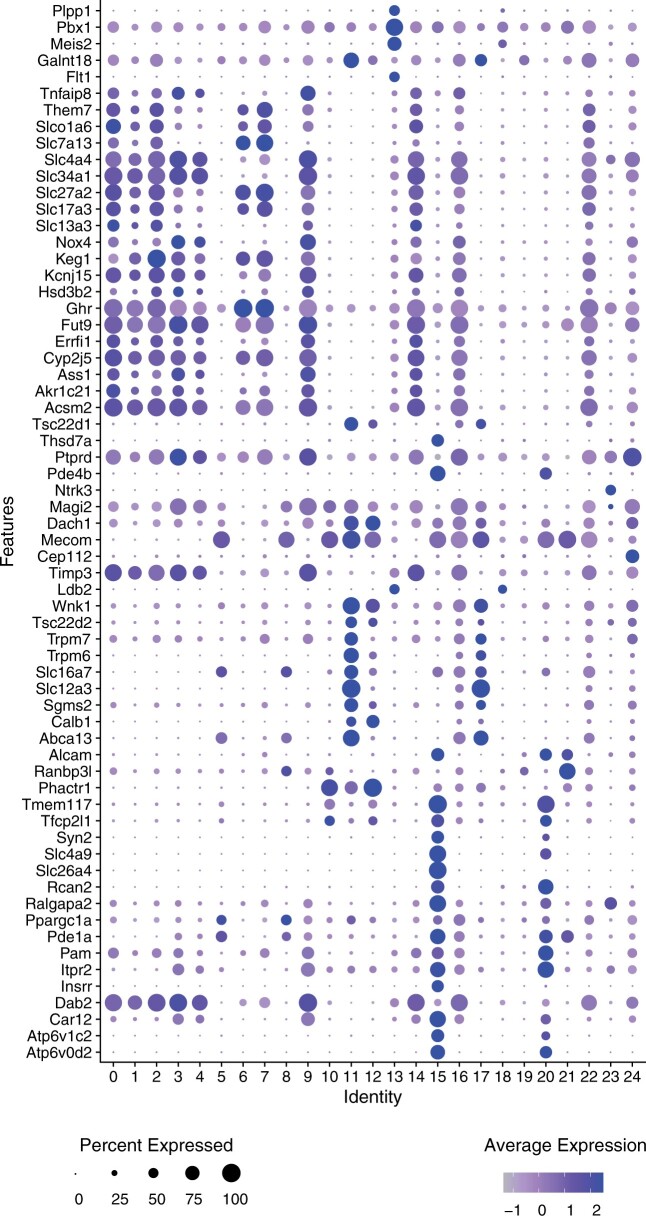
scMoC clusters marker genes. Dot plot showing the DE genes (i.e. markers) of the scMoC clusters. Expression of a set of Mouse kidney marker genes across scMoC clusters. The color intensity of the dot represents the average expression of the gene across the cells in the cluster and the size relates to the percentage of cells within the cluster expressing each gene

Figure 3 depicts a dot plot indicating the expression of marker genes in each of the clusters (Section 2). For example, *Cndp2*, *Cyp2e1*, *Keg1*, S*lc13a3*, *Ugt2b38* are DE in cluster 1 and are marker genes for *Proximal tubule cells*. Cluster 11 represents *Distal convoluted tubule cells* as its DE genes overlap with the marker genes: *Abca13*, *Calb1*, *Sgms2*, *Slc12a3*, *Slc16a7*, *Trpm6*, *Trpm7*, *Tsc22d2* and *Wnk1*. While cluster 15 is expressing *Collecting duct intercalated cell* marker genes (e.g. *Atp6v0d2*, *Atp6v1c2*, *Car12*, *Insrr*, *Ralgapa2*, *Rcan2*, *Slc26a4*, *Slc4a9*, *Syn2*, *Tmem117*). The full list of marker genes and clusters can be found in Supplementary Figure S10.

#### 3.2.2 ATAC induced splits do show differences in marker genes

The dot plot shows that some clusters have almost the same list of marker genes. For example, clusters 15 and 20. These two clusters originate from one RNA-based cluster but are split based on the scATAC-seq data. Interestingly, we do observe that there are differences in the level of the expressions of the demonstrated marker genes between the two scMoC clusters (Fig. 3, color intensity of dots). However, these differences were not sufficient to split in two clusters based on the RNA data only. But, incorporating the imputed ATAC data allows the differentiation between these two subpopulations.

#### 3.2.3 DE genes for ATAC induced splits

**Fig. 4.**
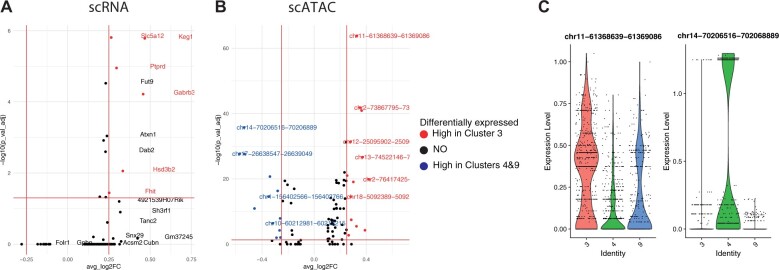
DE genes and differentially accessible peaks for ATAC induced splits. Volcano plots showing (**A**) DE scRNA genes, and (**B**) differentially accessible scATAC peaks between scMoC cluster 3 versus scMoC clusters 4&9. (**C**) Violin plot for the top upregulated peak (i.e. chr11-61368639-61369086) and top downregulated peak (i.e. chr14-70206516-70206889)

To study induced cluster splits more thoroughly, we calculated the differential expression between split clusters. Figure 4 shows the volcano plot of such a split resulting in scMoC clusters 3 and 4&9, which originally were one RNA-only cluster (cluster 0, in Fig. 2A). These clusters are categorized as Proximal tubule cell as derived from the associated marker genes (Fig. 3). But Figure 4 shows that the three scMoC clusters have a different distribution for the identified marker genes. Supplementary Figure S11 shows similar observations for other cluster splits. Supplementary Figure S12 shows the distribution of the top DE genes on scMoC cluster.

#### 3.2.4 ATAC peaks mark ATAC induced splits

To check the effect of scMoC clusters on the ATAC data, and to find whether the scMoC clusters preserve cluster differences, we calculated the differential accessible peaks for scMoC clusters 3 versus scMoC clusters 4 and 9. Figure 4B shows the difference in the accessibility of the peaks between these two clusters. We selected the top upegulated and downregulated peaks between these clusters and showed the difference in the distribution between these clusters cells (Fig. 4C), which does confirm the differences between the clusters based on the scATAC-seq data. Supplementary Figure S13 shows the distribution of the top differentially accessible peaks on scMoC clusters.

#### 3.2.5 scMoC clusters capture known motifs

To assess the biological validity for scMoC clusters, we performed an enrichment test for the clusters to search for the known motifs (Section 2). Table 2 shows the top five motifs found when comparing scMoC cluster 3 versus scMoC clusters 4&9. Four of the five motifs link to transcription factors related to long-term complications of kidney or kidney development. The MAZ transcription factor is expressed in all tissue except kidney (Bossone *et al.*, 1992) and may cause amyloid depositions in the kidney. Together confirming the role of these differential ATAC peaks to be related to differences in kidney cell types. A list of the top 20 Motifs is in Supplementary Figure S14.

**Table 2.** List of the top 5 motifs found in enrichment test for scMoC cluster 3 versus scMoC clusters 4&9

ID	Motif	UNIPROT	Disease links/function (UNIPROT/GeneCards)
MA0153.2	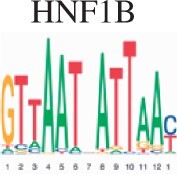	P27889	Diabetes and renal disease with small/single/horseshoe kidney, diabetes mellitus with long-term complications effecting kidney, prostate cancer.^a^
MA0039.4	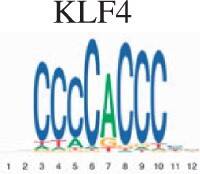	Q60793	Kidney development, pancreatic
MA0046.2	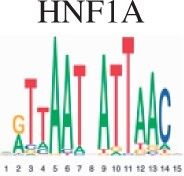	P22361	Pancreatic islet cells, liver, diabetes mellitus with long-term complications of kidneys
MA0733.1	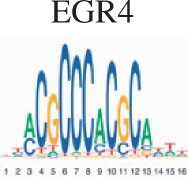	Q9WUF2	Mitogenesis and differentiation
MA1522.1	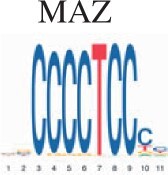	P56671	Expressed in kidney, diabetes mellitus 2 with long-term effects on kidney, amyloidosis depositing in kidney

a The function mentioned is derived from the similar motif in Homo-sapiens.

### 3.3 Applying scMoC to different protocols

#### 3.3.1 scMoC generalizes to other protocols suffering from data sparsity

**Fig. 5.**
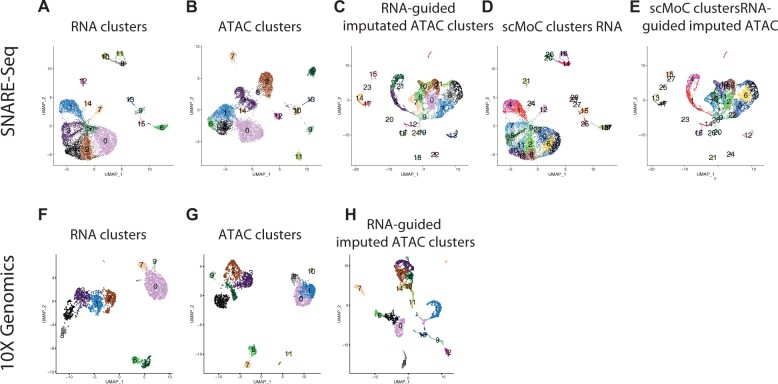
Applying scMoC to SNARE-seq and 10X genomics multiome data. UMAP visulation for SNARE-seq and 10X genmics multiome data. (**A**) SNARE-seq scRNA-seq data, colors indicating the RNA-based clusters. (**B**) SNARE-seq scATAC-seq data, colors indicating ATAC-based clusters. (**C**) RNA-guided imputed SNARE-seq scATAC-seq data, colors indicating clusters within the imputed data clustered independently. (**D**) SNARE-seq scRNA-seq data colored according to the scMoC clusters. (**E**) RNA-guided imputed scATAC-seq data colored according to the scMoC clusters. (**F**) 10X genomics multiome scRNA-seq data. (**G**) 10X genomics multiome scATAC-seq data. (**H**) RNA-guided imputed 10X genomics scATAC-seq data

To test how well scMoC generalizes to a multimodal protocol suffering from data sparsity, we applied it to SNARE-seq data that has a data sparsity of ∼99%. The RNA-guided imputation decreased the data sparsity to 77.6% (Fig. 5B and D). Combining the imputed ATAC-only clustering with the RNA-only (Fig. 5A) clustering led to new clusters (Fig. 5C and E) which were not observed either in the RNA-only or the unimputed ATAC-only analysis.

#### 3.3.2 Applying scMoC to data with matching sparsity levels

Next, we applied scMoC to 10X genomics multiome data for which the scATAC-seq data have about the same sparsity as the scRNA-seq data (both ∼93%). Figure 5F and G display the scRNA-seq and scATAC-seq data, respectively, showing that the scATAC-seq data (without imputation) has a similar clustering structure as that of the scRNA-seq data (Supplementary Fig. S15). When imputing the ATAC data using scMoC (Fig. 5H), the distribution of the cells is clearly affected.

#### 3.3.3 Defining the benefiting limits of RNA-guided imputation in scMoC

**Fig. 6.**
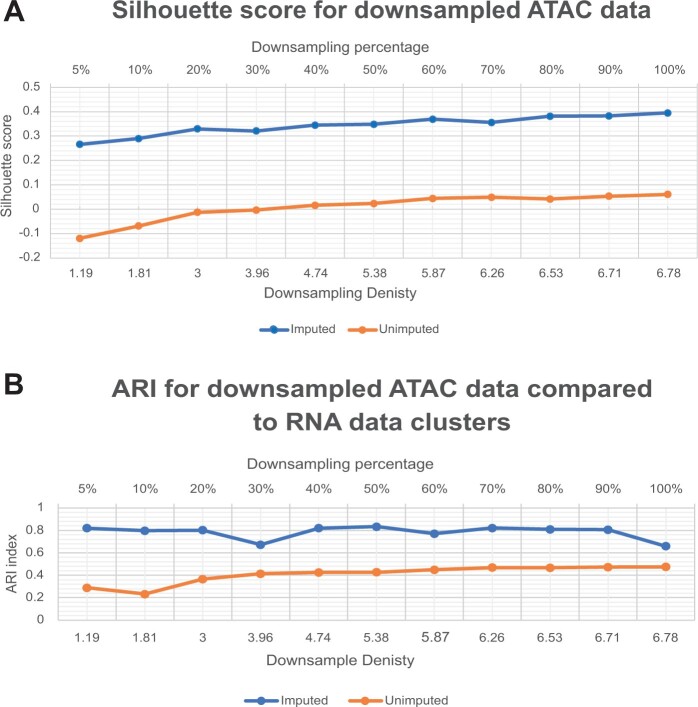
Defining the benefiting limits of RNA-guided imputation in scMoC. (**A**) Silhouette score for downsampled scATAC-seq data. Measuring the Silhouette score for the resultant scMoC clusters for RNA-guided imputed and unimputed downsampled scATAC to simulate variable data densities. (**B**) ARI for downsampled ATAC data compared to the clusters generated from the full RNA data. It measures the consistency between the RNA clusters versus the ATAC either RNA-guided imputed or unimputed data

To find the sparsity limits at which the RNA-guided imputation can help, we simulated the data sparsity by downsampling the 10X genomics Multiome data. We downsampled from 5% of the actual data and increased gradually to 100% using the *scuttle* R package (McCarthy *et al.*, 2017). To test how well the RNA-guided imputation can help in retaining the data structure, we clustered both downsampled (RNA-guided imputed and unimputed) data at each downsampling percentage using Louvain algorithm (Blondel *et al.* 2008). We assessed the quality of the resulting clusters by comparing the silhouette score of the RNA-guided imputed and unimputed downsampled versions of the data (Fig. 6A). Our results show that applying scMoC with imputation to the downsampled data consistently results in a higher silhouette score over the whole range of data densities. Also, it can be noticed that the downsampling only moderately and gradually affects the clustering. Note that the silhouette score of the unimputed data is much lower across the whole range, which might be caused by the local smoothing introduced by the imputation as well as that we find less clusters in the imputed setting, both positively affecting the silhouette score.

To further assess the RNA-guided imputation of scMoC, we compared the clusters found in the imputed and unimputed downsampled ATAC data with the clusters found when using only the RNA data (Fig. 6B). These results show that the RNA-guided imputation helps increasing the cluster agreement between the two different modalities.

To measure the cluster agreement between imputed and unimputed ATAC data, we calculated the adjusted R and index (ARI) between the unimputed ATAC data without downsampling, as common ground, and the RNA-guided imputed and unimputed downsampled ATAC data. Supplementary Figure S16A shows that as the data density increases the RNA-guided imputation generated clusters become more alike the unimputed ATAC which explains the difference in the Silhouette score. Furthermore, we quantified the preservation of the local neighborhood of cells. Hereto, we calculated the Jaccard index between the 50 nearest cells in the data without downsampling with the neighborhood of that cell after downsampling (with and without RNA-guided imputation). From Supplementary Figure S16B, we can observe that the neighborhood is preserved better using the RNA-guided imputation when the data density becomes lower than 3%. Hence, the data sparsity affects more the local neighborhood than the clustering and the RNA-guided imputation can recuperate the loss of information when the data density becomes below 2–3% in ATAC compared to 7% in RNA data. Although this seems low, the sci-CAR and SNARE-seq data densities are even much lower by which they are 0.28% and 1.01%, respectively (see Table 1).

To further assess the ability of the RNA-guided imputation to recover the ATAC signal in the downsampled data, we used the RNA-guided imputed ATAC data without downsampling as the common ground and calculated the ARI to both downsampled (RNA-guided and unimputed) data. Supplementary Figure S17 shows that the imputation can recover most of the signal compared to the RNA-guided imputed ATAC data and it performs better than the unimputed version. Also, it shows that the imputation method is consistent on the different ranges of data sparsity.

#### 3.3.4 scMoC sensitivity and accuracy

To check the sensitivity of scMoC to the selection of the neighborhood window size (*k*), we calculated the silhouette score across different settings ranging from 10 to 100 neighbors when applying on the 10X-genomics dataset. Supplementary Figure S18 shows that the Silhouette has a consistent performance for the RNA-guided imputation strategy across all tested values of neighborhood sizes, with a small gain at around the 50 neighbors.

To assess the accuracy of the imputation scheme, we compared the imputed downsampled version of the 10X-genomics data to the unimputed original data. Supplementary Figure S19 shows the root mean square error (RMSE) for different downsamplings. The RMSE values are consistent over the range of downsampling percentages, except when downsampling to 5% or 10% of the data.

## 4 Discussion

We have shown the ability of scMoC to cocluster data from scRNA-seq and scATAC-seq measured from the same cell. scMoC’s imputation strategy made it possible to exploit the sparse scATAC-seq data. scMoC’s joint clustering scheme of both data domains revealed biologically meaningful clusters that are supported by the expression of known cell-type-specific markers and splits induced by the ATAC data are related to motifs for which transcription factors are related to the tissue of consideration. An important contribution of scMoC is its imputation strategy: it uses the less data-sparse modality (here, the scRNA-seq data) to impute the data-sparse modality (here, the scATAC-seq data). This leverages the fact that both modalities are measured at the same cell and thus that the other data modality can be used to find similar cells. We stress that scMoC is built upon the assumption that the local neighborhood of a cell as derived from the less-sparse omic data is a better approximation of the cell neighborhood than the neighborhood determined from the sparser data. In other words, that cells in different omics domains have a similar local neighborhood. This is, however, also a limitation as different modalities are representing different types of information and as such might result in different neighborhoods, e.g. two subpopulations of cells might only differ in the transcriptional profiles but not in their accessibility profiles. Our experiments, however, show that the assumption of equal neighborhoods is valuable.

We have shown that the usage of scMoC is beneficial when used on different data densities However, missing the ground truth made it hard to quantify the actual gain of using the imputation. The scMoC imputation strategy can be extended to other multi-omics protocols that suffer from sparsity. According to the philosophy of our imputation strategy, one can search for the neighborhood in less-sparse data and then impute the sparse data based on the scMoC assumption of preserving the local neighborhood of the cell in different modalities. Another limitation is the usage of the KNN search algorithm that is scaling quadrable with the data, however, an extension to scMoC is to use more efficient KNN search algorithms (e.g. based on kd-trees).

With our work, we have highlighted that single-cell multimodal measurements are a valuable tool to resolve heterogeneity at the single-cell level, even if the data sparsity of one of the modalities is high.

## Funding

European Union’ H2020 research and innovation program under the MSCA grant agreement [861190 (PAVE)]; European Commission of an H2020 MSCA award [675743] (ISPIC); NWO Gravitation project: BRAINSCAPES: A Roadmap from Neurogenetics to Neurobiology (NWO: 024.004.012).

*Conflict of Interest*: none declared.

## Supplementary Material

vbac011_Supplementary_Data
