# Supplementary material for: scMoC: single-cell multi-omics clustering
Source: Bioinform Adv. 2022 Feb 15;2(1):vbac011. doi: 10.1093/bioadv/vbac011 (PMC9710707; doi:10.1093/bioadv/vbac011)
Supplement: vbac011_Supplementary_Data [file vbac011_supplementary_data.zip › Supplementary_figures_revised.pdf]

Supplementary

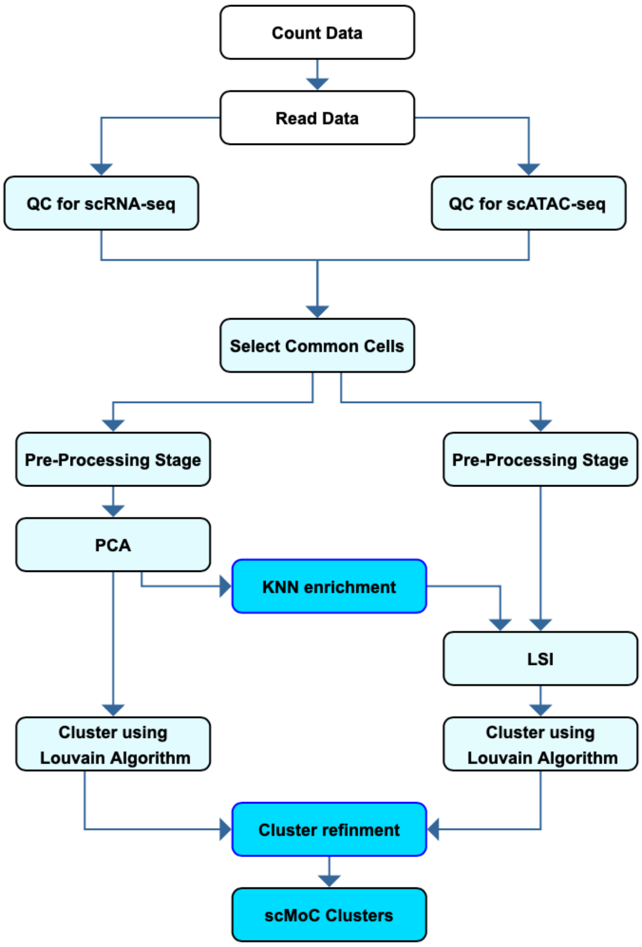

Supplementary Figure 1: scMoC block diagram scMoC applies the Seurat V3 pipeline in processing the RNA data. First the Data undergoes a quality control step. Then the cells that have both RNA and ATAC data measured and passed the previous step is selected to go through the rest of the steps. For the RNA, the data undergoes the pre-processing stage of Normalizing the data and then scaling up by factor 1e4. The Data is then projected to the PCA space. Afterwards, the data RNA data is clustered. For the ATAC data after the pre-processing stage, the neighborhood of each cell is searched in the PCA projected space of the RNA. The KNN enrichment step is to take the average of the peak values for each cell. After that the data is renormalized and projected to the LSI space to be clustered. After clustering both data domains the cluster refinement step is made to get the splits of the ATAC in the RNA cluster and then he non-assigned cells from the original RNA cluster are assigned to the closest cluster based on the average distance to the cells within a cluster using Euclidean distance in the RNA space only.

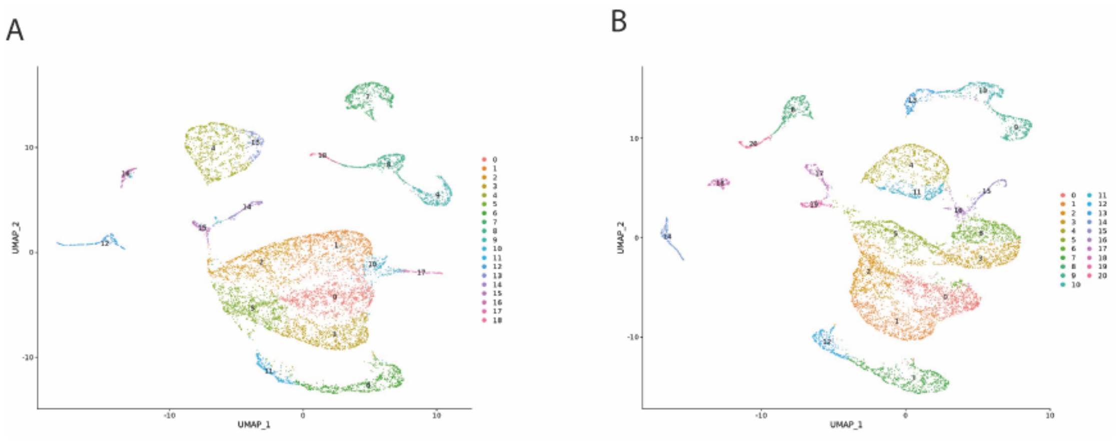

Supplementary Figure 2: Studying the effect of different dimensionality reduction algorithms over the sci-CAR RNA-guided imputed data. (A) using the PCA reduction method. (B) using the LSI reduction method. Both panels using the top 20 vectors.

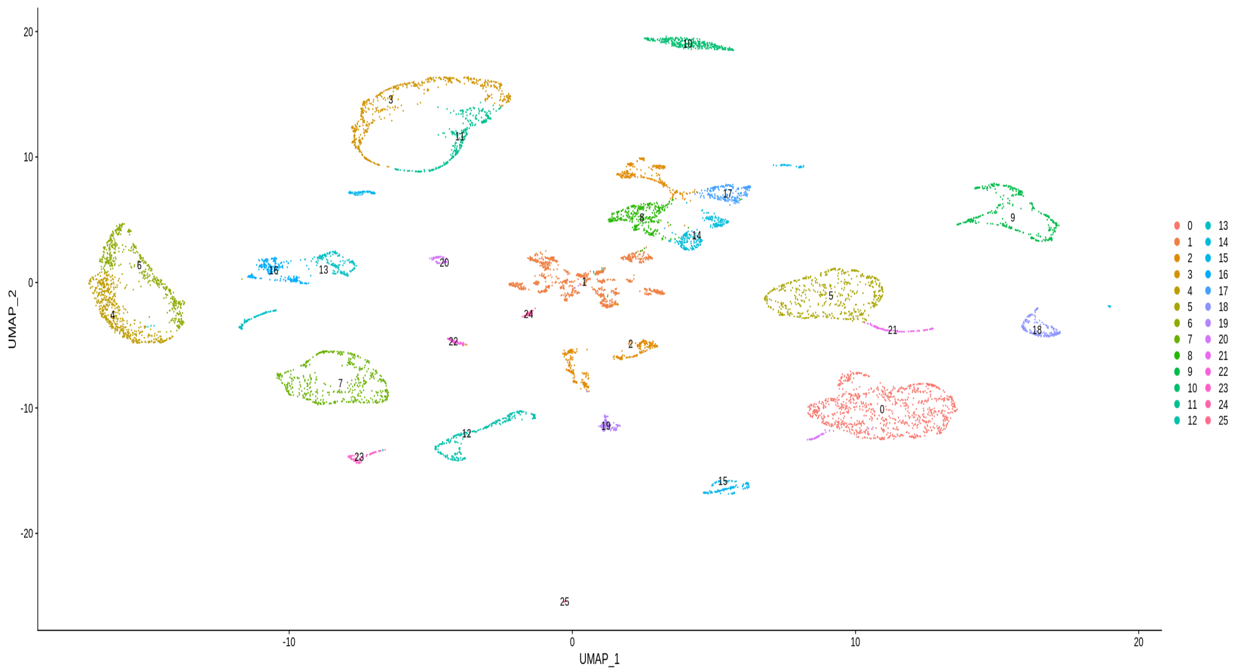

Supplementary Figure 3: UMAP for the sci-CAR scATAC-seq data after using *RNA-cluster guided imputation* when the selection of the neighbors is limited to cells from matching RNA cluster. The UMAP shows deformation in the scATAC-seq clusters, e.g., by the appearance of line structures.

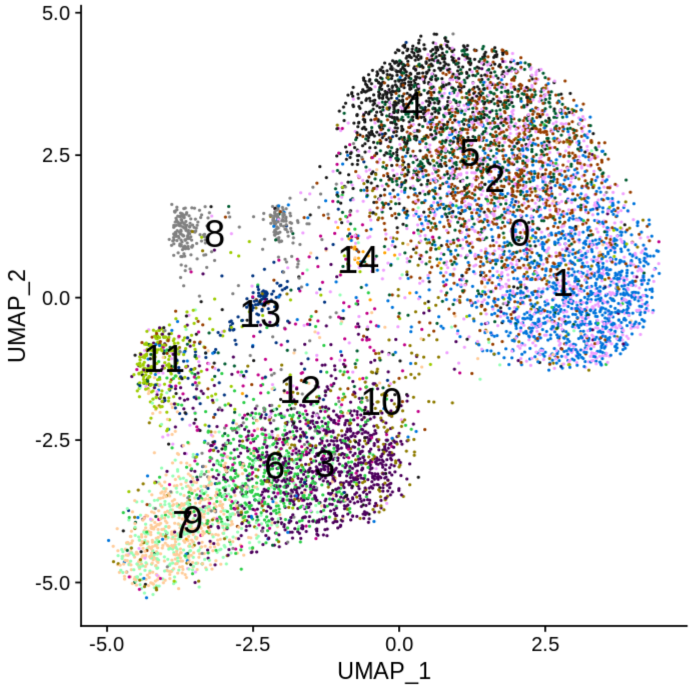

Supplementary Figure 4: sci-CAR scATAC-seq data colored with the scRNA-seq clusters showing a discrepancy between both clustering in both data domains

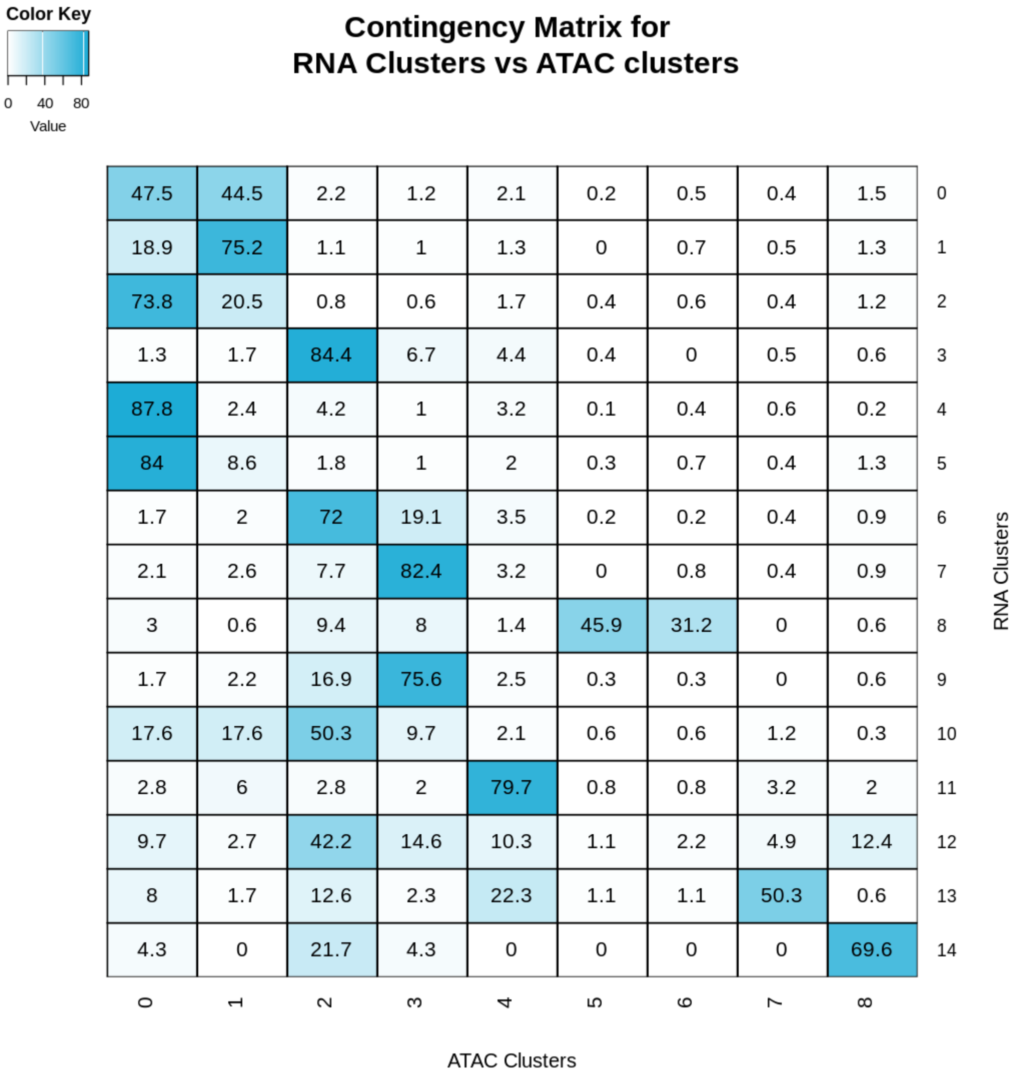

Supplementary Figure 5 : Contingency Matrix between the scRNA-seq and scATAC-seq without imputation (clustering resolution = 0.8).

Contingency Matrix for
RNA Clusters vs imputed ATAC clusters

RNA Clusters \ imputed ATAC Clusters	0	1	2	3	4	5	6	7	8	9	10	11	12	13	14	15	16	17	18	19
0	0.4	1.7	47.5	3.2	0.2	46.7	0	0	0	0	0	0	0.3	0	0	0	0	0	0	0
1	0.5	4.7	0	54	36.9	3.8	0	0	0	0	0	0	0	0	0	0	0	0	0	0
2	76.1	1.7	12.4	2.1	7.2	0.2	0.3	0	0	0	0	0	0	0	0	0	0	0	0	0
3	0	0	0	0	0.3	0.2	0	54.3	44.3	0	0	0	0	0	0.1	0.8	0	0	0	0
4	0.3	0.8	0.1	0	0.2	0	68	0	0	0	0	0	30.5	0	0	0	0	0	0	0
5	1.4	94.7	3.3	0.3	0	0	0.3	0	0	0	0	0	0.1	0	0	0	0	0	0	0
6	0	0	0	0	0	0.2	0	0.2	0.4	65.5	0	0.2	0	0	33.4	0	0	0	0	0.2
7	0.4	1.1	0.4	0.6	2.8	0.6	0.2	0.4	0.9	0	66.6	0	0	0	0.2	0	0	0.4	0	25.5
8	0	0.6	0.3	0	0.3	0	0	0	0	0	0	0	0	56.9	0	0	0	0.3	41.7	0
9	0	0	0.3	0	0	1.1	0	0	0	3.7	2	92.7	0.3	0	0	0	0	0	0	0
10	9.4	10.6	2.4	5.2	37.9	5.2	2.7	7.9	5.2	7.6	0	3	0.3	0	2.7	0	0	0	0	0
11	2.4	4	2.8	4.8	10.8	5.6	1.2	0	0	0	0	0.4	0.8	0	0.4	66.5	0	0	0.4	0
12	0	0.5	0	0.5	14.1	1.6	0	0	0	0.5	0	0	0	0	0	0.5	1.1	80.5	0	0.5
13	1.7	2.3	0.6	0.6	0.6	0.6	1.1	0.6	1.1	0	0	0	0	0	0	0.6	90.3	0	0	0
14	0	0	0	4.3	91.3	0	0	4.3	0	0	0	0	0	0	0	0	0	0	0	0

Supplementary Figure 6 shows the Contingency Matrix between the scRNA-seq and scATAC-seq with RNA guided imputation. This matrix is used to decide on the splitting and refinement of the scMoC clustering. When there is an agreement between the clusters between 10% and 90%.

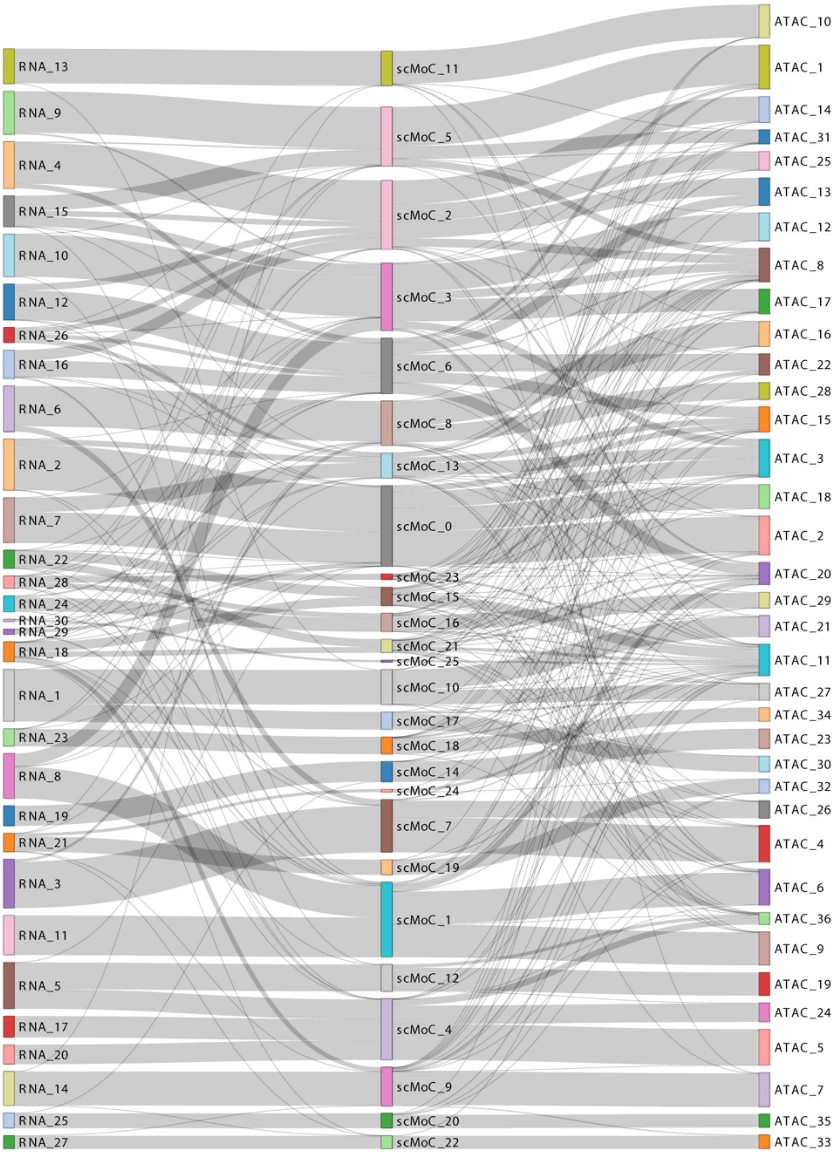

Supplementry Figure 7: Sankey graph showing how the scMoC clustering relates to the scRNA-seq and (imputed) scATAC-seq based clusterings. The middle panel shows the detected scMoC clusters. To show that the ATAC-based splits induced (of the scRNA-seq clustering) are not similar to a more fine-grained clustering of the RNA data. The left panel shows the RNA-based clusters when using a more detailed resolution (so more RNA clusters). Same holds for the right panel showing the fine-grained clusters of the ATAC data. In some cases (e.g., the top scMoC cluster 11), the more fine-grained RNA clustering did not split the scMoC cluster, as can be noticed that scMoC cluster 11 completely overlaps with the more fine-grained RNA cluster 13. For scMoC cluster 1 (scMoC_1), we see that it is split into clusters RNA_8 and RNA 11 when we cluster at a more fine-grained level. We also observe that this splitting matches a more fine-grained clustering of the ATAC data, that is RNA cluster RNA_14 matches the more fine-grained ATAC cluster ATAC_7 and they agree with scMoC cluster 9 (scMoC_9).

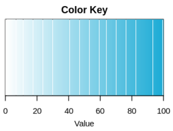

Contingency Matrix for
RNA Clusters vs ATAC clusters

1	2	3	4	5	6	7	8	9	10	11	12	13	14	15	16	17	18	19	20	21	22	23	24	25	26	27	28	29	30	31	32	33	34	35	36	37	RNA Clusters
0.6	0	0	0	0	0	0.3	0	0	0	0	0	82.1	100	0	0	0	0	0	0	0	0	0	0	0	0	0	0.7	0	0	0	0	0	0	0	0	0	1
0	0	0	0	88.5	0	0	0.6	0	0	1.3	0	0	0	0	0	0	0	37.9	1.3	0	0	39.8	0	0	4.4	0	0	0	0	0	0	0	0	11.4	0	0	2
76.2	0	0	0	0	0	0	0	0	0	1.3	0	0	0	0	0	45.9	0	0	0	0	0	0	0	0	0	0	0	0	0	0	0	0	0	0	0	0	3
0	5.6	0	0	0	0	0	0	77.3	54.4	0	3.4	0	0	0	0	0	0	0	0	0	0	0	0	0	0	0	0	0	0	0	0	0	0	0	0	0	4
0	0	0.3	0	0	1.7	0	0	0	0	0.3	0	0	0	0	0	0	0	0	0.8	100	78.6	1	0	0	40.5	0	0	0	0	0	0	0	0	0	0	0	5
12.5	0	0	0	0	0	0	0	0	0	0	0	0	0	0	0	50.4	0	0	0	0	0	0	0	98.9	0.6	0	1.3	0	0	0	0	0	0	0	100	0	6
0	0.7	1.1	72	0	0	0	0	0	0	0.6	0	0	0	0	0.4	0	4	55	0	0	0	21.4	0	0	0.6	0	0	0	0	0	0	0	0	0	0	0	7
0	1	78.2	1.4	0	0	0	2.6	0	0	0.3	0	0	0	0	0	0	48.8	0	5.9	0	0	2.9	0	0	5.1	0	0	0	0	0	0	0	0	0	0	0	8
0	2.9	0	0	0	0	0	0	0	14.7	0	0	0	0	82.1	5	0	0.4	0	0	0	0	0	0	0	1.9	0	87.3	0	0	0	0	0	0	0	0	0	9
0	0.2	0	20.9	0	0	0	0	0	0	0.3	0	0	0	0	93.9	0	31.3	0	0	0	0	0.5	0	0	8.2	0	0	0	0	0	0	0	0	0	0	0	10
0	0	12.4	0	1.1	0	0	92	0	0	0	0	0	0	0	0	0	0	0	9.7	0	0	3.4	0	0	1.3	0	0	0	0	0	0	0	0	0	0	0	11
0	0.7	0	0	0.5	0	0	0	15.7	0	1.6	74.4	0	0	0	0	0	0	0	2.1	0	0	0.5	0	0	0	0	0	0	0	0	0	0	0	76.2	0	0	12
0	0	0	0.3	0	0	1.4	0	0	0	0.3	0	7.6	0	0	0	0	0	0	0	0	0	0	100	0	0.6	96.7	1.3	0	0	0	0	0	0	0	0	0	13
1	0	0	0	0	0	93.5	0	0	0	0.3	0	0.3	0	0	0	0.7	0	0	0	0	0	0	0	0	0.6	0	0	0	0.7	2.9	0	0	0	0	0	0	14
0	34.1	1.6	0.3	0	0	0	0	0	23.4	1.3	0	0	0	17.1	0.7	0	13.1	0	0	0	0	0	0	0	2.5	0	0	0	0	0	0	0	0	0	0	0	15
0	21	3	0	0.3	0	0	1.1	3.5	0	0.3	9.1	0	0	0	0	0	0	0	58.2	0	0	4.4	0	0	0	0	0	0	0	0	0	0	0	2.9	0	0	16
0	0	0	0	0.3	54.3	0	0	0	0	0.6	0	0	0	0	0	0	0	0	0	0	5.8	0	0	0	4.4	0	0	0	0	0	0	0	0	0	0	0	17
0	1.2	0.8	1.1	2.5	0.8	4.2	0.3	1.7	1.5	24.5	4.7	9.7	0	0	0	0	0.8	0	6.3	0	0.5	2.9	0	0	4.4	2.6	0	0	0	0	0	0	0	1	0	0	18
0	0	0	0	0	0	0	0	0	0	0.6	0	0	0	0	0	0	0	0	0	0	0	0	0	0	0.6	0	0	0	0	0	0	99.3	0	0	0	100	19
0	0	0	0	0	42.9	0	0	0	0	0	0	0	0	0	0	0	0	0	0	0	14.6	0	0	0	8.9	0	0	0	0	0	0	0	0	0	0	0	20
0	0.2	0	0	0	0	0	0	0	0	12.3	0	0.3	0	0	0	0	0	0	0	0	0	0	0	0	0	0	3.3	0	97.9	2.1	0	0	0	0	0	0	21
6.6	1.7	1.3	1.6	2.7	0	0	0	0.9	2.7	17.6	4.7	0	0	0	0	0.7	0.4	1.7	7.2	0	0	3.4	0	1.1	6.3	0	0	0	0	0	0	0	0	1.9	0	0	22
0.6	0.2	0.3	0.3	0.3	0.3	0	0	0	0	3.5	0.3	0	0	0	0	0	0	0	1.7	0	0	1.5	0	0	0	0	2	100	0.7	0	0	0	0.7	1	0	0	23
2.3	0	0	0	0	0	0	0	0	0	4.1	0	0	0	0	0	2.2	0	0	0	0	0	0	0	0	0	0.7	4	0	0	0	0	0	94.1	0	0	0	24
0.2	0.2	0	0.3	0	0	0.3	0	0	0	2.8	0	0	0	0	0	0	0	0	1.3	0	0	0	0	0	0	0	0	0	0.7	0	100	0.7	0	0	0	0	25
0	28	0	1.1	0	0	0	0	0.6	0.9	0.3	0	0	0	0	0	0	0	2.1	0	0	0	9.7	0	0	0	0	0	0	0	0	0	0	0	3.8	0	0	26
0	0	0	0	0	0	0.3	0	0	0	0.6	0	0	0	0	0	0	0	0	0	0	0	0	0	0	0	0	0	0	0	95	0	0	0	0	0	0	27
0	2.2	1.1	0.3	1.1	0	0	1.1	0.3	2.4	17	3	0	0	0.7	0	0	1.2	0.8	1.7	0	0.5	1.5	0	0	8.2	0	0	0	0	0	0	0	5.1	0	0	0	28
0	0	0	0.5	2.5	0	0	2.3	0	0	1.3	0	0	0	0	0	0	0	2.5	3.8	0	0	7.3	0	0	0	0	0	0	0	0	0	0	0	1.9	0	0	29
0	0	0	0	0.3	0	0	0	0	0	6.9	0.3	0	0	0	0	0	0	0	0	0	0	0	0	0	0.6	0	0	0	0	0	0	0	0	0	0	0	30

ATAC Clusters

Supplementary Figure 8 : Contingency table for the sci-CAR data between scRNA-seq and scATAC-seq data clustered with higher level of resolution in the clustering algorithm to generate double the number of clusters in the scRNA-seq data. This shows that there is no one-to-one agreement between the clusters.

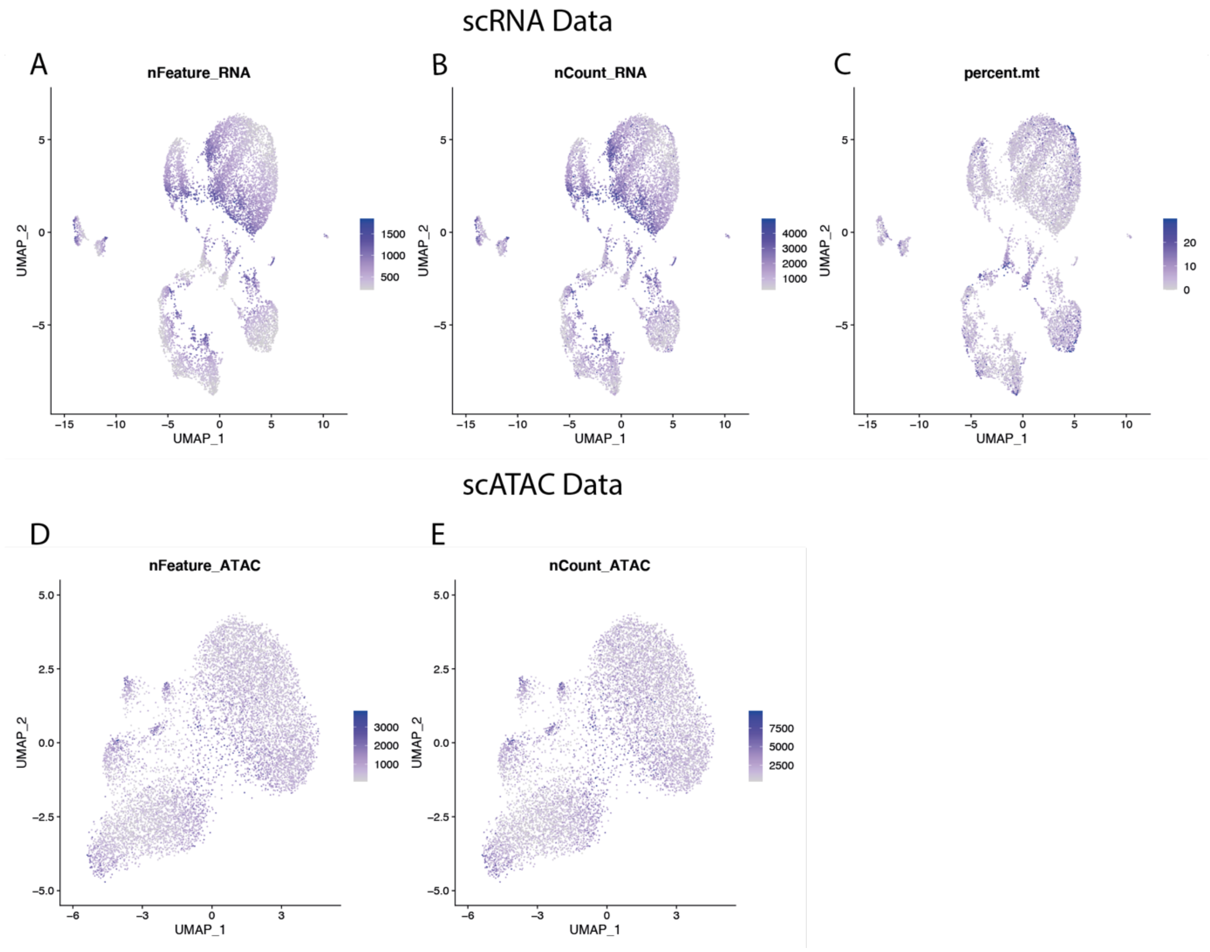

Supplementary Figure 9: UMAPs for sci-CAR data overlayed with the QC matrices. (A) scRNA-seq UMAP overlayed with the number of genes detected per cell. (B) scRNA-seq UMAP overlayed with the genes count per cell. (C) scRNA-seq UMAP overlayed with the percentages of the mitochondrial genes detected per cell. (D) scATAC-seq UMAP overlayed with the number of peaks detected per cell. (E) scATAC-seq UMAP overlayed with the count of peaks detected per cell.

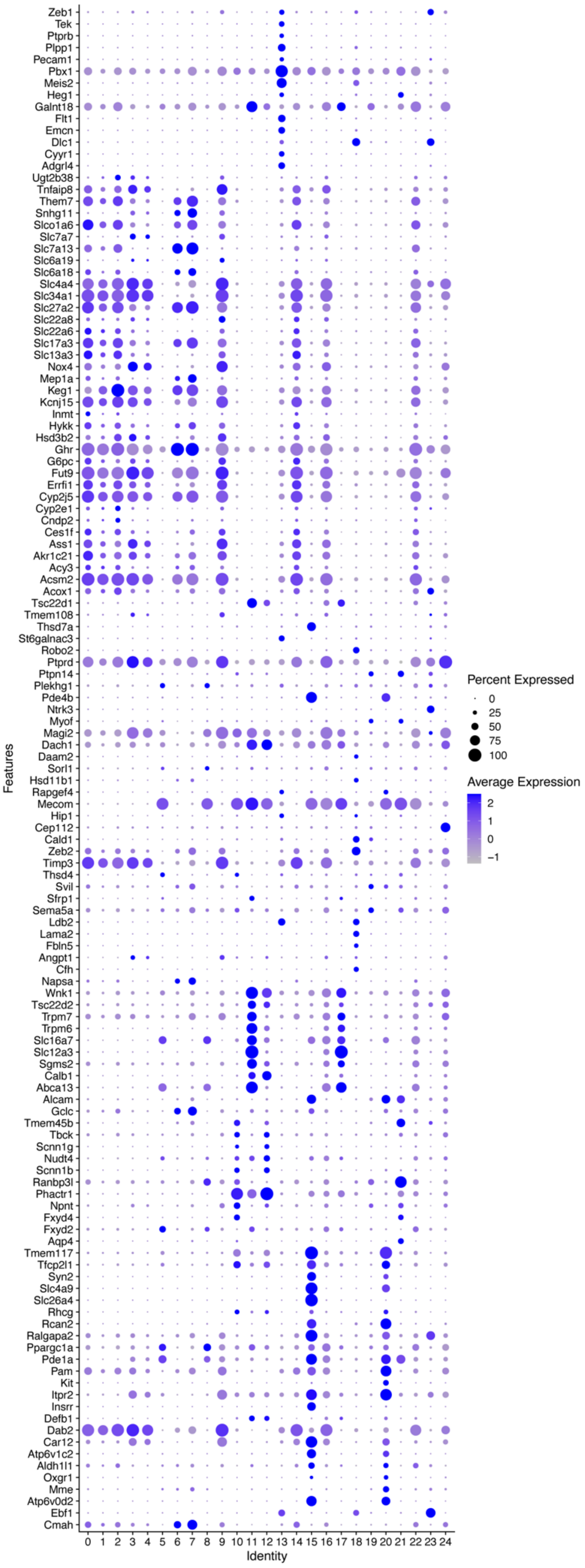

Supplementary Figure 10: Full list of the marker genes found in the scMoC clusters.

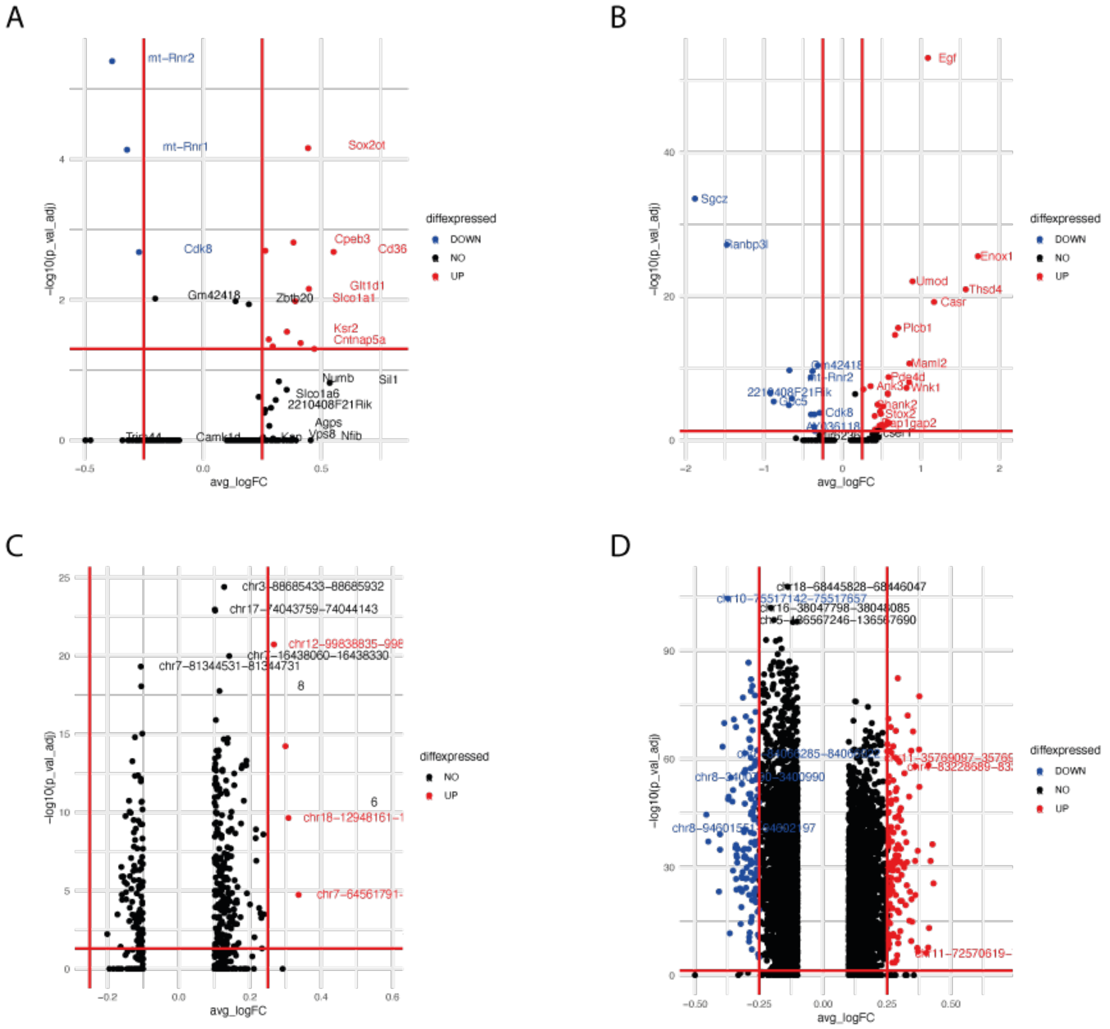

Supplementary Figure 11 : Volcano plots showing the differentially expressed genes between (A) cluster 0 vs 14 , and (B) clusters 5 vs 8. (C) and (D) are the volcano plots showing the differentially expressed peaks between cluster 0 vs 14 and 5 vs 8 respectively.

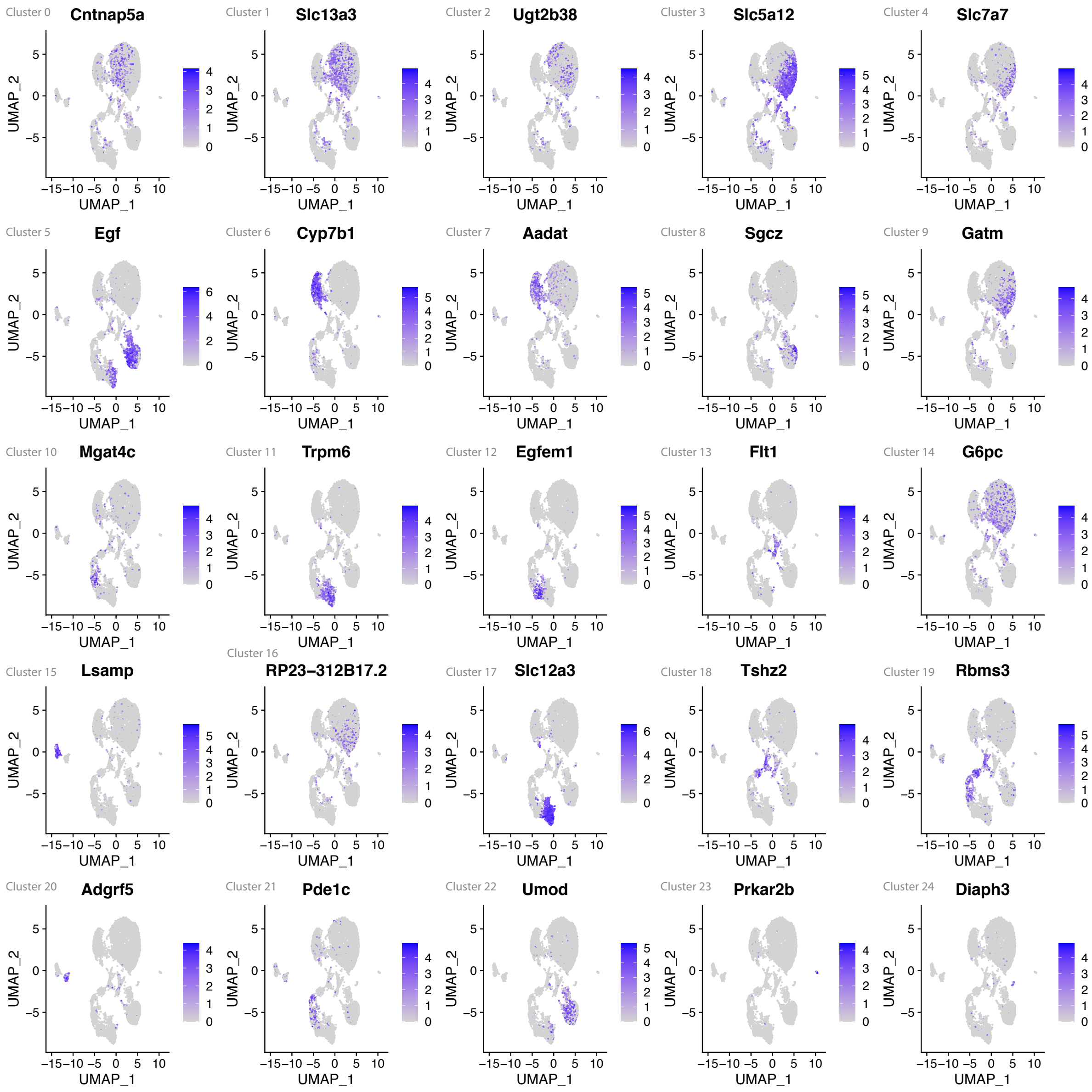

Supplementary Figure 12: Top differentially expressed genes for scMoC clusters, showing the localization of the gene expression to the newly created clusters.

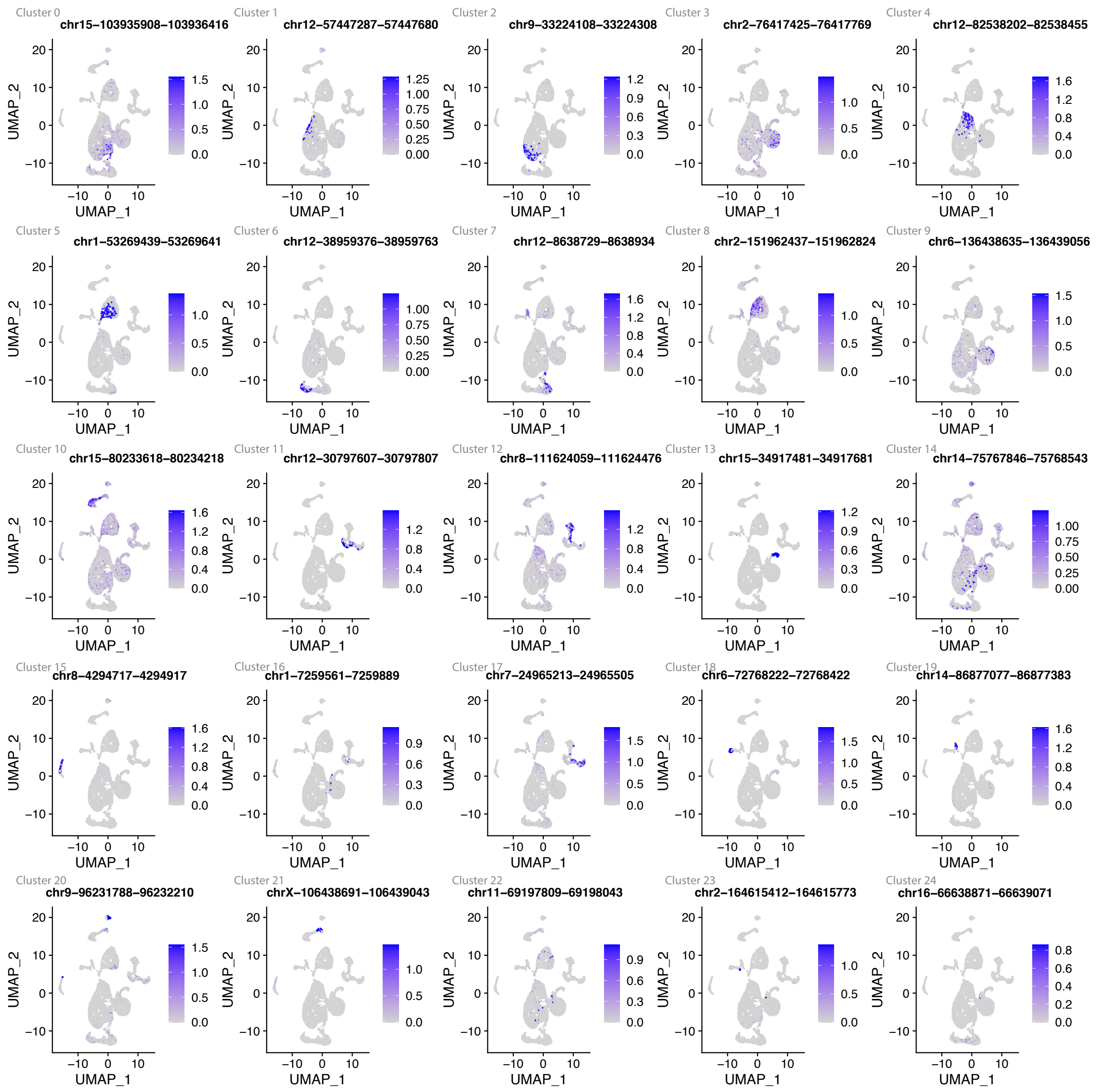

Supplementary Figure 13: Top differentially accessible peaks for scMoC clusters, showing the localization for the peak accessibility to the newly created clusters.

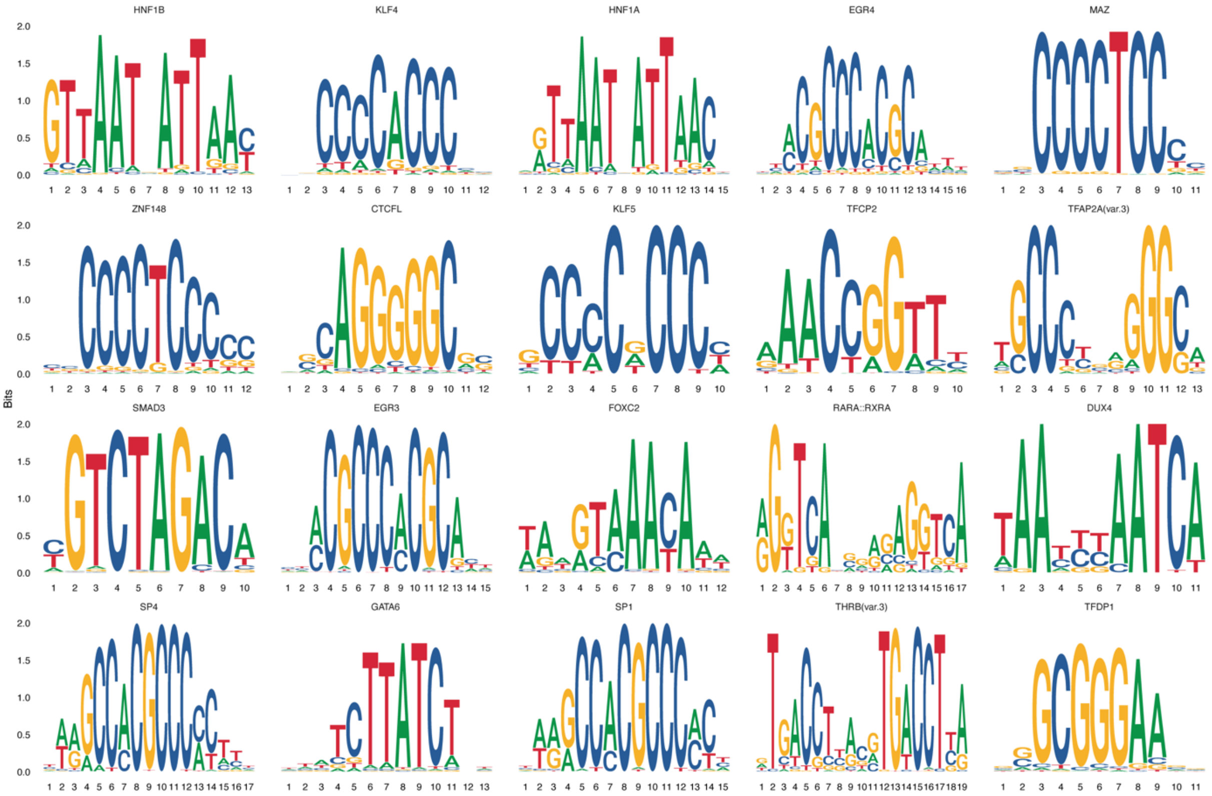

Supplementary Figure 14: List of the top 20 motifs found in enrichment test for scMoC cluster 3 vs scMoC clusters 4 & 9

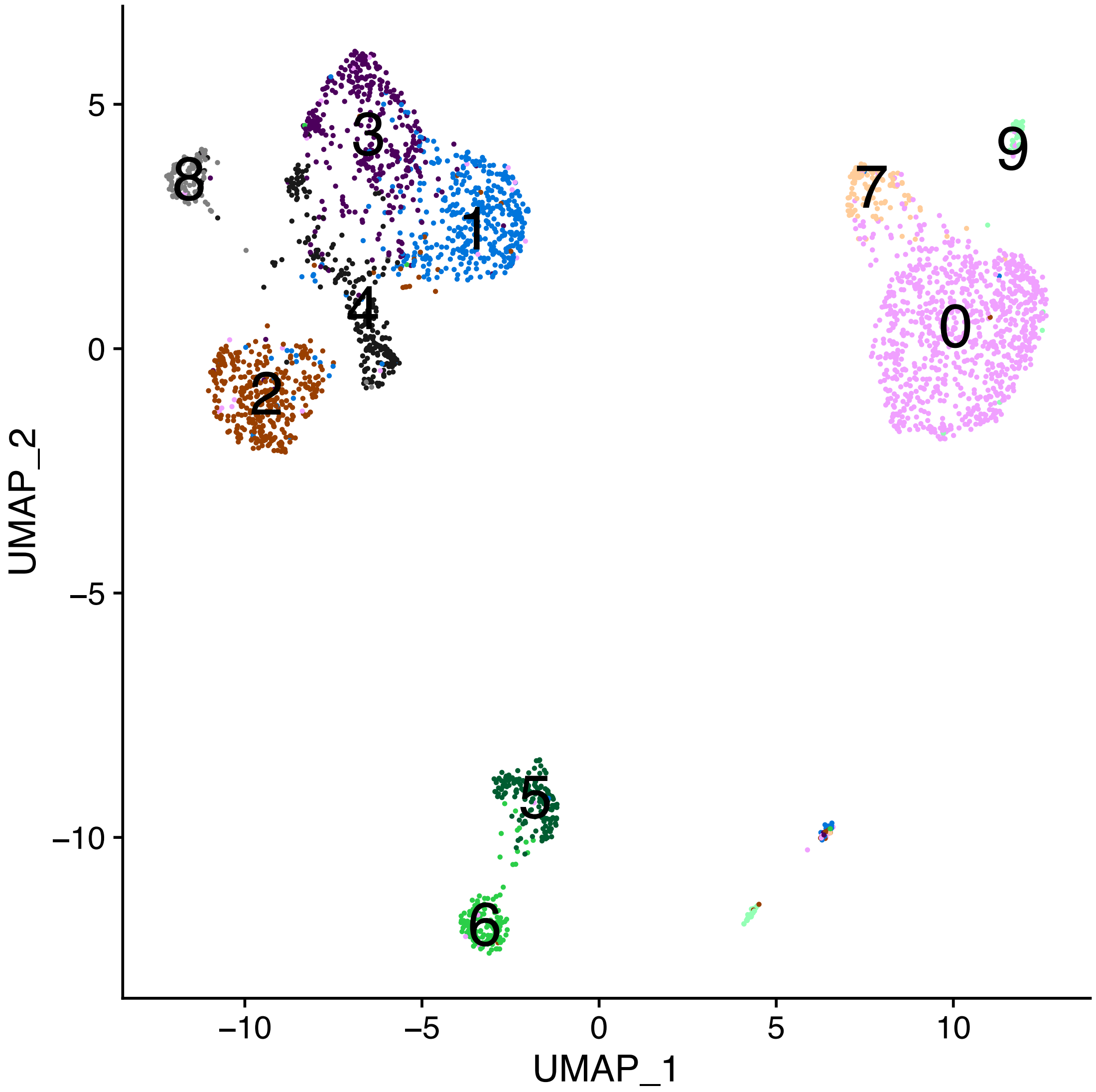

Supplementary Figure 15: The 10X genomics multiome data unimputed ATAC cluster overlaid with the RNA clusters. The graph shows the corresponds between the two data domains. In this case the effect of applying scMoC is minimized.

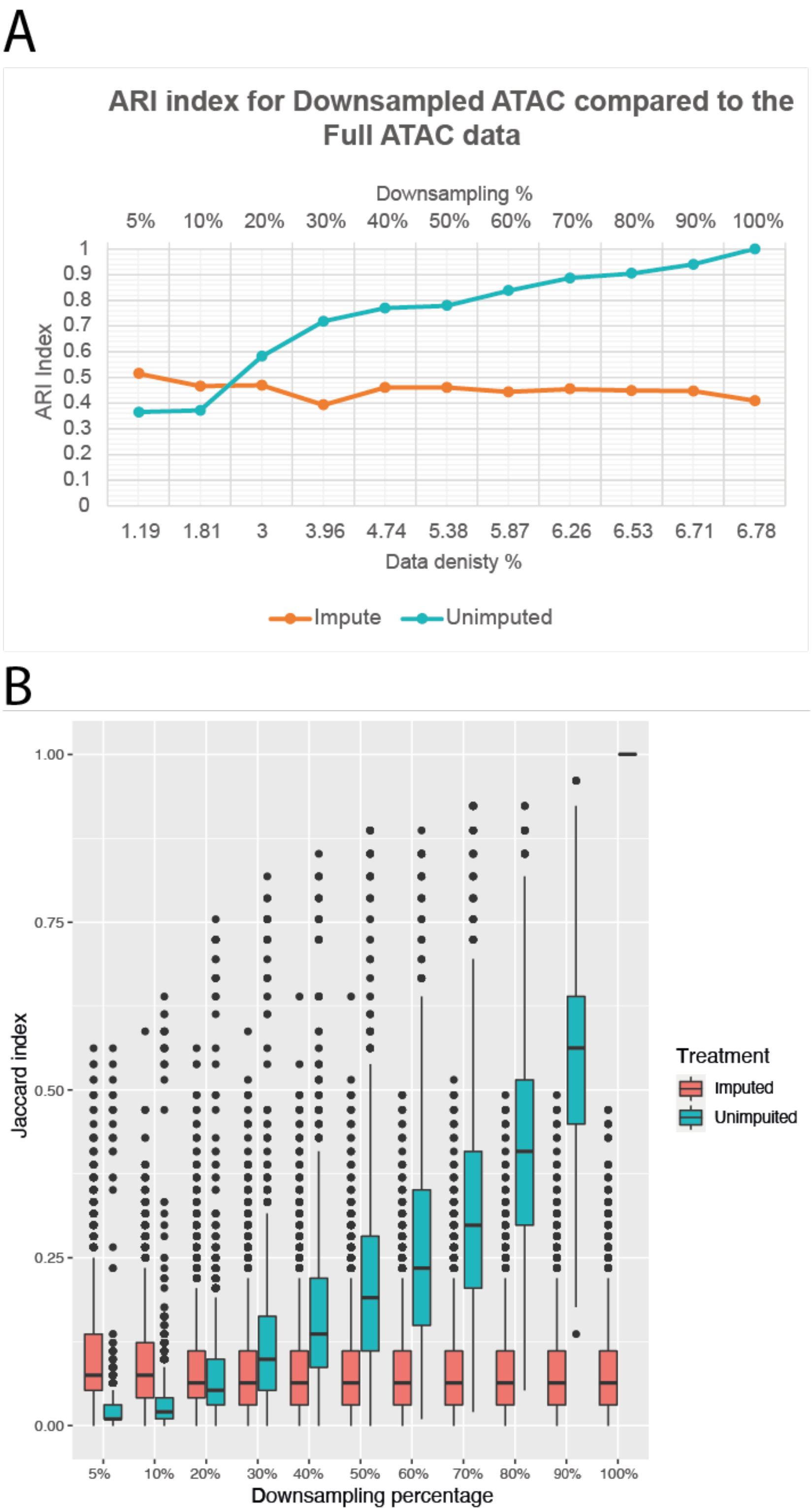

Supplementary Figure 16: Downsampling scATAC-seq data. (A) Cluster agreement (measured by the Adjusted Rand Index, ARI) between the scATAC-seq data clustered originally (100% level) and data clustered at downsampled version using either no imputation (Unimputed) as well as the proposed RNA-guided imputation (Imputed). (B) Boxplot of the neighborhood agreement between the cells in the original data (100%) and the imputed and unimputed downsampled data, measured using the Jaccard index between the 50 nearest neighbors of each cell.

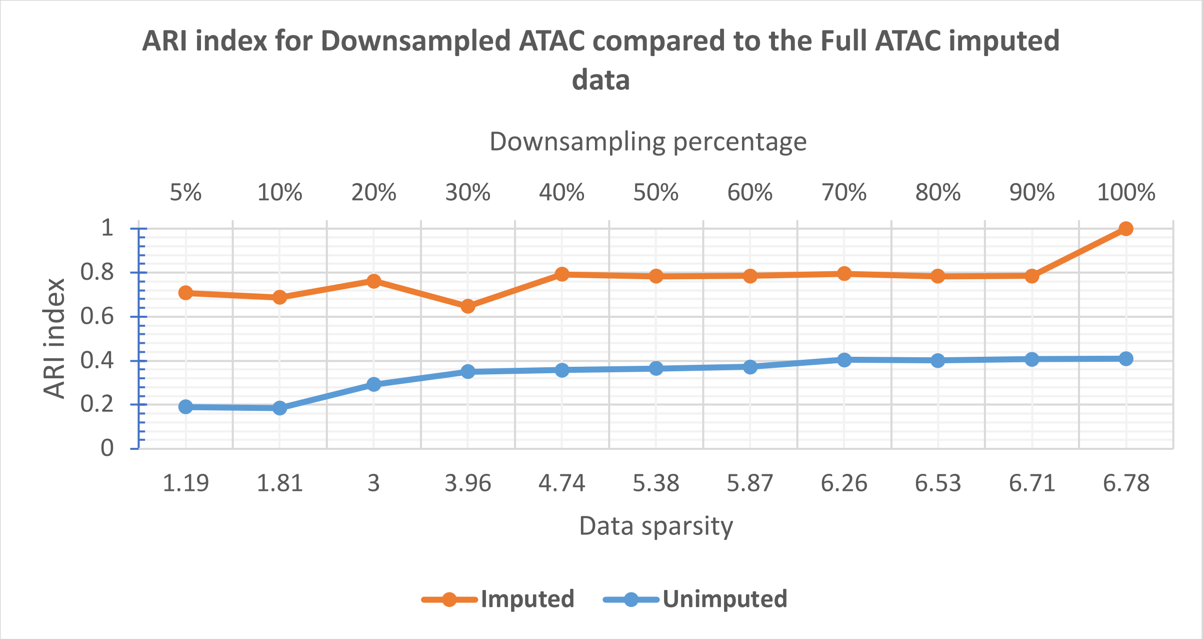

Supplementary Figure 17: Data recovering measured by ARI between the RNA-guided imputed scATAC-seq data clustered originally (100% level) and data clustered at downsampled version using either no imputation (Unimputed) as well as the proposed RNA-guided imputation (Imputed).

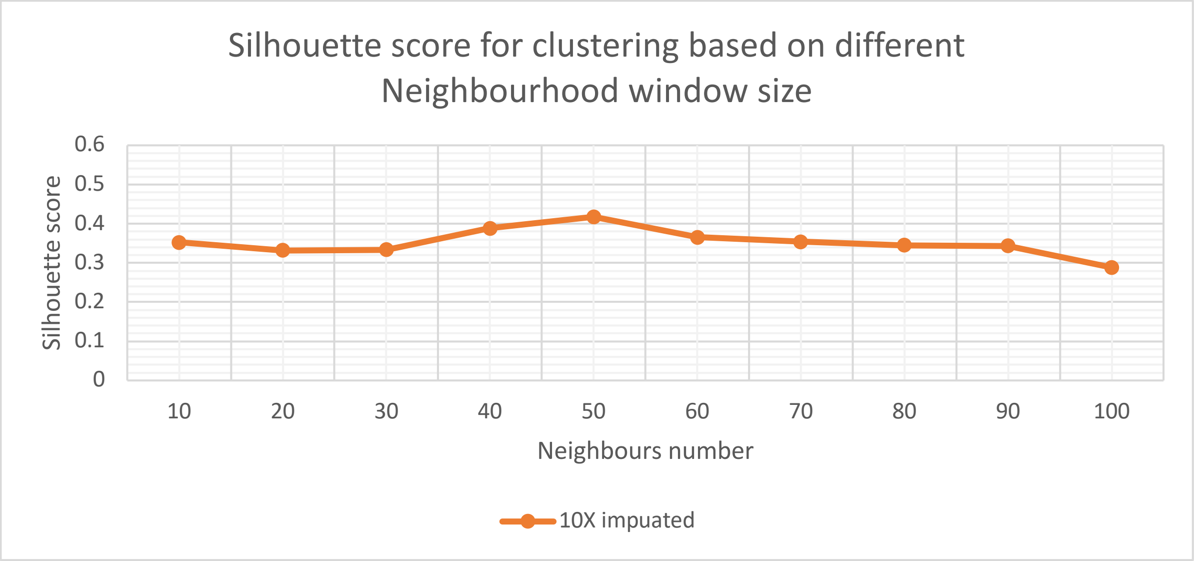

Supplementary Figure 18: Silhouette score for 10X genomics dataset imputed with different window size. The imputation tends to be consistent with the different size, with a small a peak value at the K=50

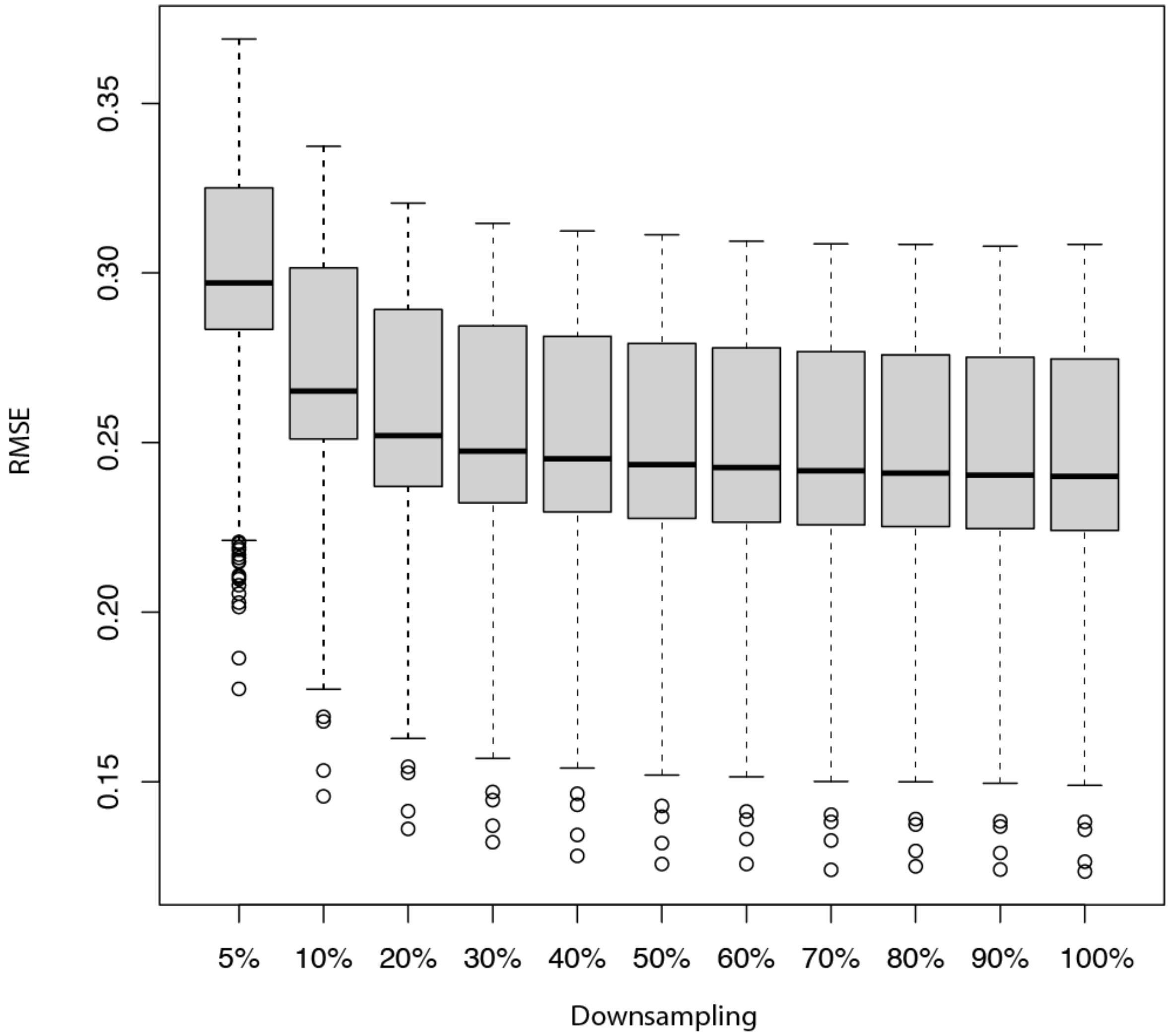

Supplementary Figure 19: Root mean square error (RMSE) calculated for the downsampled imputed 10X geneomics data compared to the unimputed 100% of the data to check the accuracy of the imputation.

Supplementary Table 1: Summarizing the limits used in processing different datasets. *Min genes per cell* defines the almost empty cells, by which cells having less than that threshold of genes is removed. The upper level of genes detected per cell so that the cell is not noisy is set by *Max genes per cell*. *Max Mito percentage* is the maximum number of mitochondrial genes to be accepted in the cell. *Min cells per genes* is set to remove genes that are detected in cells less than this threshold. *Min peaks per cell* and *Max peaks per cell* are the lower and the upper limit of peaks detected in each cell respectively. *Min cells per peak* is set to remove peaks that are detected in cells less than this threshold.

	sci-CAR	SNARE-seq	10X genomics
Min genes per cell	200	200	100
Max genes per cell	2500	2500	5500
Max Mito percentage per cell	30%	5%	30%
Min cells per gene	3	3	3
Min peaks per cell	4000	4000	200
Max peaks per cell	10000	10000	32500
Min cells per peak	3	3	3

Supplementary Table 2: Cells passing the QC limits. The table shows statstics about the available cells in each dataset and the number and the percentages of cells passed the QC step. The percentage of cells increase by the increase of the protocol quality and maturity.

Dataset	Measured Cells	Cell passes QC	QC passed %
sci-CAR	11,296	9,080	80.4%
SANRE-seq	10,309	8,890	86.2%
10X-genomics	3,003	2,920	97.2%
